# The Experience of Self-Compassion in Church of England Working Clergy: An Exploratory Qualitative Pilot Study Conducted in England

**DOI:** 10.1007/s10943-025-02373-9

**Published:** 2025-07-04

**Authors:** Belinda Norrington, Nicola Douglas-Smith

**Affiliations:** School of Education and Social Sciences, University of the West of Scotland, Paisley, UK

**Keywords:** Compassion, Self-compassion, Clergy, Church of England

## Abstract

Self-compassion improves a range of psychosocial outcomes and can support working populations experiencing burnout. Clergy can experience higher levels of burnout, but there is limited research exploring the benefits of self-compassion for this population. This qualitative pilot study, conducted in England, utilised semi-structured interviews to examine how Church of England clergy perceive, value and experience self-compassion. An inductive Qualitative Content Analysis produced three categories: ‘Compassion is deeply rooted in theology’, ‘Self-compassion is primarily practical self-care’, and ‘Self-compassion requires a surrounding ‘habitus of compassion’. This suggests clergy perceive self-compassion as practical care and with less theological validity compared to compassion for others. Participants indicated interest in self-compassion teaching/training, alongside the need for self-compassion to exist within a wider institutional culture of compassion.

## Introduction

Church of England clergy are a subset of a much larger total number of ministers of religion across multiple denominations and faith traditions that serve their local communities in England. Latest figures put the number of Church of England clergy at approximately 20,000 (Research & Statistics, 2021), serving close to a million regular churchgoers across every parish in the country (Eames, 2023). They work in a highly vocational context with diverse and challenging responsibilities, including provision of practical care and spiritual comfort to the distressed and dying (Terry & Cunningham, 2020, 2021). Research shows this is not without some personal cost to clergy well-being, (Edwards et al., 2020; Hendron et al., 2014). Due to the paucity of self-compassion research involving Church of England Clergy and understanding that this professional population are employed in large part to offer unstinting compassion to others, a pilot study was proposed to explore this topic further. This pilot study seeks to qualitatively explore how self-compassion is understood and experienced by a small sample of Church of England clergy in relation to their well-being and working life. Furthermore, the pilot study approach allows for the testing of the utility and validity of the chosen recruitment protocol, analysis design and data collection methods (a key strategy proposed by Malmqvist and colleagues (2019)) to facilitate high quality design of subsequent more substantive studies.

### Conceptualisations of Self-Compassion

Neff (2003, 2011) and Gilbert (2015, 2017, 2021) provide in-depth psychosocial definitions of self-compassion. Neff (2003) describes self-compassion as being ‘open to one’s own suffering, not avoiding or disconnecting from it, generating the desire to alleviate one’s suffering and to heal oneself with kindness’ (p.87). Neff’s (2003) model of self-compassion identifies three necessary capacities: self-kindness (versus self-judgment), a felt sense of common humanity (versus isolation), and mindful awareness (versus over-identification with thoughts and feelings).

Alongside Neff’s conceptualisation of self-compassion, Gilbert provides evolutionary insights to this topic which supports our understanding of self-compassion, emotional regulation and well-being (Gilbert, 2015, 2017, 2021). Gilbert (2015) highlights that self-compassion sits within a wider experience of compassion which is oriented in three possible directions: from self to others, from others to self, and from self to self. In common usage today, compassion is generally agreed to signify: ‘Sensitivity to the suffering of self and others, with a deep desire to relieve that suffering and its causes’ (HH Dalai Lama cited in Choden & Regan-Addis, 2018, p.145). Self-compassion is located, therefore, in this third ‘towards self’ flow of compassion described by Gilbert (2015, 2017). In his theory of Emotion Regulation Systems, Gilbert suggests that self-compassion supports well-being due to supporting the regulation of emotions via de-activation of the threat system with its limbic responses, and activation of the self-soothing system (Gilbert, 2017). Neff’s and Gilbert’s theoretical models bring together biological, psychological and social factors of self-compassion, and provide the conceptual framework for self-compassion for the current study.

### Self-Compassion and Well-Being in the Workplace

Germane to this research is a growing number of reports suggesting self-compassion may buffer some of the effects of work-related stress and burnout and increase components of well-being across a range of working population samples (Anjum et al., 2022; Dodson & Heng, 2022; Kirby et al., 2017). Much of this research has focussed on emergency, health, social care and teaching professions, due in part to the high rates of work-related stress, burn-out and compassion fatigue (Conversano et al., 2020; Garcia-Carmona et al., 2019).

Clergy roles and caring responsibilities have inherent demands such as unpredictable working hours, crisis management and complex safeguarding responsibilities that compare closely to those of professionals in social work, health and teaching (Adams et al., 2017). Research in the field of clergy well-being, unsurprisingly therefore, point towards significant and often debilitating levels of work-related stress, vicarious trauma and burnout (Adams et al., 2017; Francis et al., 2018; Grosch & Olsen, 2000; Hendron et al., 2014). Indeed, a recent paper by Smith et al (2025) stated that 35% of Church of England curates in their study sample experienced ministry-related emotional exhaustion in their second year of work. This was seen to be present despite concurrently having high levels of job satisfaction.

### Support for Clergy Within the Church of England

The need to support clergy well-being has been recognised for some time by the Church of England itself, resulting in the commissioning of an ongoing ten-year longitudinal study in clergy well-being (the ‘Living Ministry’ research programme) and the pioneering Clergy Covenant for Wellbeing project (Butler, n.d.). However, The Church of England have not fully considered the possible role of self-compassion training to support their clergy’s well-being. This is despite publishing a short article on their website (Clergy Covenant Working Group, n.d.) quoting findings of Adams et al. (2017) and Barnard and Curry (2012) that self-compassion has a role to play in clergy resilience and flourishing. Instead, practical self-care and faith-based practices are recommended (Clergy Covenant Working Group, n.d.). Although there is extant research elsewhere investigating the effectiveness of faith-based coping mechanisms for clergy well-being (Brewster, 2014; Pargament, 2001), it is not known how much clergy utilise faith-based practices, such as prayer and worship, to either meet, complement or replace their need for self-compassion.

### Locus of Control in Clergy

This issue may tap into theories of Locus of Control (Ng et al., 2006; Padmanabhan, 2021). If the Locus of Control for clergy is externally driven by factors such as the transformational power of religious tradition/practices, sacred texts and relationship with God, it may be that internally self-generated compassion is less easily understood, valued or trusted for reasons unique in substance or degree for religious professionals. This study hopes to address this knowledge gap.

### Church of England Clergy and Self-Compassion

This paucity of research examining self-compassion as a specific psychological phenomenon of potential relevance to clergy well-being has not been restricted to the Church of England. Unlike the rapidly growing number of scientific studies investigating the protective effects of self-compassion in similarly demanding secular professions (Hashem & Zeinoun, 2020; Neff et al., 2020), significantly less has been undertaken for clergy (Edwards et al., 2020). Where research has been undertaken in this area, higher self-compassion has been associated with less work-related stress and burnout (Barnard & Curry, 2011; Lee & Rosales, 2020). Most of these studies are focussed on clergy in the USA (Barnard & Curry, 2011, 2012; Lee & Rosales, 2020; Parker, 2020), with scant research in clergy-based self-compassion for those working in the Church of England context. This is important because there are thousands of different Christian denominations within/across different geographical and theological contexts which have significant variance regarding role expectations and demands. It follows therefore, that findings for one denominational group cannot necessarily be extrapolated straightforwardly across the wider clergy populations without the risk of overly homogenised conclusions.

### Current Study

The primary purpose of this pilot study is to provide a preliminary qualitative exploration and elucidation of key elements of the personal experience and perceptions of self-compassion in the working lives of a sample of currently employed Church of England clergy. The broader purpose is to identify the most persistent commonalities in how Church of England clergy understand and experience the phenomenon and how they feel the cultivation of self-compassion might be useful in supporting their well-being and ministry, or conversely, ways in which working clergy think there may be difficulties/downsides to cultivating self-compassion. Not only will this exploratory study add to the scant literature available on this topic, but by piloting the qualitative research methods and design (Malmqvist et al., 2019), it will offer scope for honing the research instruments used for maximum efficacy, thereby addressing study weaknesses at an early stage of research and laying the ground for further higher quality research examining the value of self-compassion for this population. The through line of purpose in present and future stages of research will be both rigorous and ethical investigation of the phenomenon of clergy self-compassion and a high-utility contribution to the well-informed care of clergy well-being in the Church of England.

## Methods

### Design

This was a small-scale, explorative, qualitative pilot study investigating how currently employed Church of England clergy understand, value and experience self-compassion, addressing the knowledge gap in the literature for this topic.

### Ethics

The study was guided by BPS ethical guidelines and ethical approval was obtained by University of the West of Scotland’s Education and Social Science Ethics committee (reference number: 2024–22557-17666).

### Participants

Inclusion criteria for recruitment included working as a member of Church of England clergy, in the roles of parish priest/vicar, curate or chaplain. Using convenience sampling within a highly specific population group, seven members of Church of England clergy based in England were successfully recruited—a satisfactory number for a small-scale qualitative study (Terry et al., 2017) (Table 1).

**Table 1:** Participant Demographics

Participant	Presenting Gender	Role	Length of Interview
A B C D E F G	Female Female Female Male Female Male Female	Parish priest Parish priest Parish priest Parish priest Parish priest Parish priest Curate (priest in training)	30 m 02 s 30 m 18 s 39 m 50 s 63 m 11 s 48 m 25 s 48 m 29 s 36 m 37 s

### Materials and Procedure

Study procedure followed the Bengtsson protocol (2016), employing the specified phases of Planning, Data Collection, Data Analysing, and Report Creation and Presentation. Data were collected over a few consecutive weeks during April/May 2024, via one-to-one, semi-structured interviews, one per participant, lasting 30–60 min. A total of ten main questions, informed by the relevant psychological literature on compassion (Gilbert, 2017, 2021; Neff, 2003, 2011), were pre-determined but open-ended. Probe questions were used where appropriate to achieve maximum data collection. Interviews were conducted and recorded online via Microsoft Teams, and the auto-generated transcripts were downloaded by the researcher. Each transcript was fully anonymised to ensure confidentiality and anonymity for participants, as per BPS ethical guidelines (British Psychological Society, 2021).

### Analysis Procedure

Data were subjected to inductive Qualitative Content Analysis (Bengtsson, 2016; Elo & Kyngäs, 2008), with the purpose of identifying, collating and reporting the most frequent patterns of manifest meaning related to the research question. The analytical process followed a procedure laid out by Bengtsson (2016), which is broken down into four phases: Decontextualisation, Recontextualisation, Categorisation and finally Compilation. Colleague checking was employed at two stages (the early stages of the open coding and during the categorisation stage) to support the credibility/rigour of the analysis (Bengtsson, 2016).

Although a quantitative measure code frequency took place, this can only be described as a quantitative analytical step in the basic sense of counting and collating codes to identify key categories. Since no further statistical analysis was conducted, the analytical procedure remained overall qualitative rather than mixed method. This approach was adopted because it was best suited to centring participants’ subjective and contextual experience of the phenomenon.

## Results

**Fig. 1:**
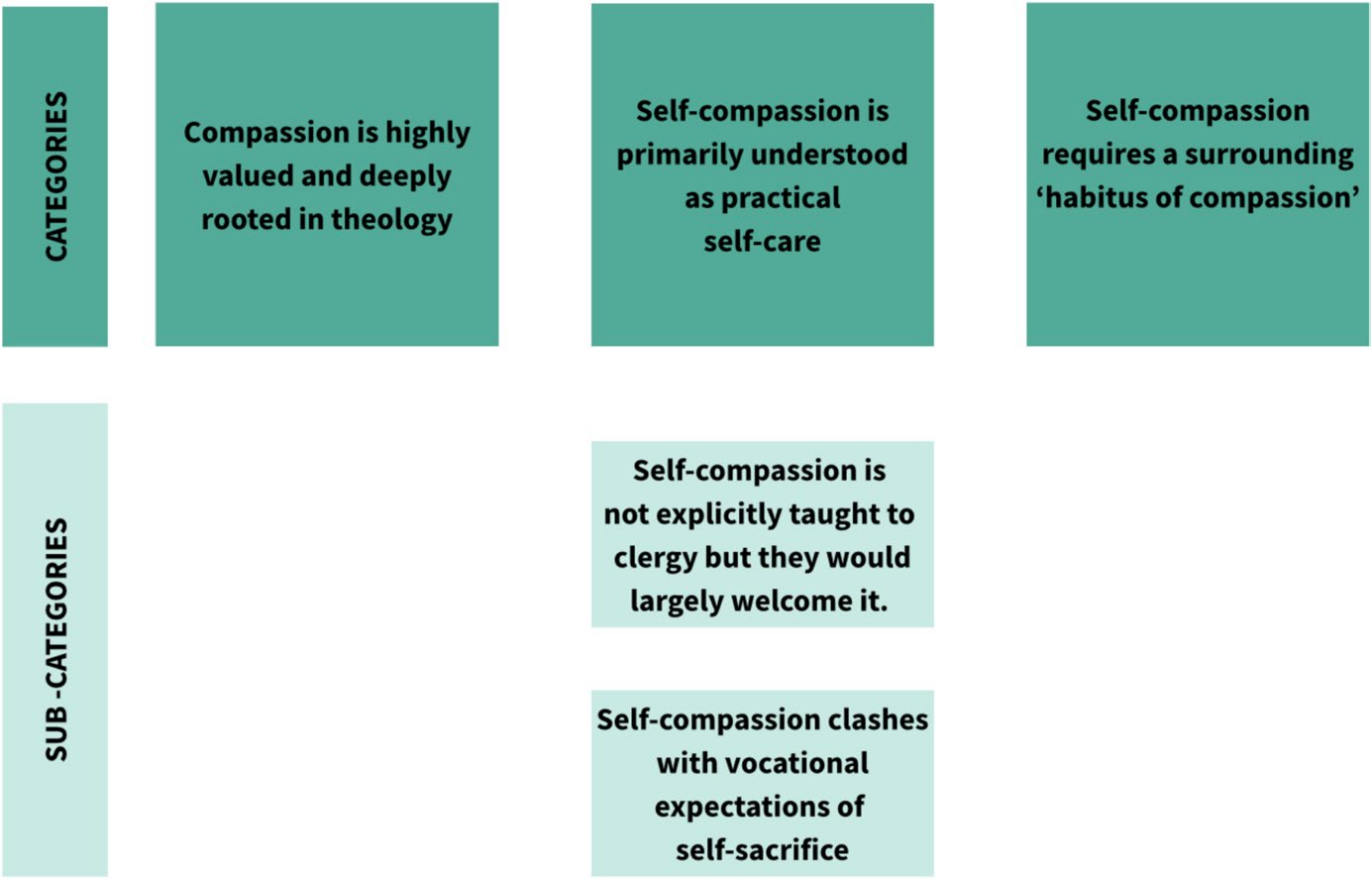
Thematic Categories and sub-categories

Results across three categories together represented the most pressing issues and commonalities found in the data regarding how participants understand, value and experience self-compassion. The categories and sub-categories can be seen in Fig. 1.

### Compassion is Highly Valued and Deeply Rooted in Theology

Within this category, key findings were contained in high-frequency extracts (interview quotes) spread across every transcript. Since this analysis model prioritises only the most frequent commonalities in the data, the less persistent codes are not discussed in this paper. ‘Theological’ at a basic descriptive level simply means ‘related to the study of religion and religious beliefs’ (Cambridge University Press, n.d.), but for this specific sample, the term signifies the study/understanding of Christian and Protestant beliefs specifically.

When asked how they understand compassion, participants conveyed a richly faith-based perspective. Participant D was representative of this view: “*… that love that I believe for me is from another source impacts my own life so, yeah, I think that’s initially where I start with compassion*” (25–26). As most did, he referred to biblical sources for evidence: “*the two ultimate commands, love God and love your neighbour as yourself… they’re not options* (875–876 and 882). This demonstrates an external motivation for love/compassion, in which an obedient response to God is highly valued. Indeed, in one biblical reference given, John 15:10 (*Holy Bible (NIV),* 2008), Christ says those that disobey his command to love others cannot remain in His love; a stark, externally controlled outcome (and potential motivator), for this highly religious sample.

There was also a clear sense of compassion being an action-based behaviour. This quote summed up the pattern of responses given by participants to explain compassion’s active quality: “*it’s not—sympathy, it’s more got empathy involved, but there’s probably more action involved than just empathy? So, it involves kind of taking steps to be compassionate.*” (Participant G, 25–27). These insights reflect psychological literature on the topic (Gallagher et al., 2024; Neff, 2003, 2023), particularly the empathic connecting to suffering/sufferer, rather than remaining separate and superior which has been identified as part of the interpersonal dynamic of sympathy and pity (Gerdes, 2011).

However, there was nuance regarding the necessity of action within compassion since some clergy stressed that simply being present and prayerful witness to suffering constitutes being compassionate. As Participant F said, “*It could just be the appreciation, and the presence, and that could be the response”* (43). *I think that compassion has two strong elements. One is the active one…one is an embodiment*” (201–202). It should be noted that the ultimate template for both the action-based compassion and more quietly embodied expression was still largely rooted in theology, as first-and-foremost being modelled by Christ: “*He kind of …just kind of entered to where they were and walked with them through things. And so I think, yeah, as a Christian, it’s a really important value*” (Participant G, 68–71). This quote succinctly sums up this category’s overall message from participants, that compassion is deeply grounded in theology and required and modelled by Christ in ways that emphasise embodied, caring presence to suffering rather than pitying or fleeing from it.

Within this category, participants pointed to the commonly felt and highly valued benefit of compassion to relationship building: “*I think it’s an emotional connection*” (Participant C, 134). Compassion can therefore be understood to be highly valued in different ways by the sample, spiritually, emotionally and relationally.

### Self-Compassion is Primarily Understood as Practical self-care

This category’s key findings identified differences in how clergy understand, and experience self-compassion compared to compassion for others. The data presented self-compassion as primarily understood as practical self-care for the protection of well-being as Participant F said: “*It needs to look like two simple things, sleep and silence’* (329). A common practical example given was creating diarised free-time blocks to decompress from the rigours of ministry work: “*…there doesn’t seem to be any spaciousness at the moment in my week and that’s not very good for me and that I need to try and block that in.*” (Participant E, 588–589).

There were only six manifest examples of priests placing self-compassion in a faith-based context. This may partly be explained by the first half of ‘Sub-category: Self-compassion is not explicitly taught to clergy, but they would largely welcome it’.

### Self-Compassion is not Explicitly Taught to Clergy, but they Would Largely Welcome it

High frequency extracts for this sub-category evidenced a lack of explicit teaching about self-compassion in clergy training, the focus instead being on self-care for well-being. Participant A’s views were representative: “*I don’t think it was ever described as kind of how to have self-compassion or that sort of thing….like a specific session or anything like that on it”* (233–235). This lack of teaching may plausibly be one reason there is a relative lack of theological depth to clergy perceptions of self-compassion. When asked in interview if self-compassion training might be of interest or not, clergy largely responded that they would be interested. Participant A suggested self-compassion training might help clergy feel permitted to attend kindly to their own needs; “*I think a lot of it is about that kind of permission stuff, which I think, uh, yeah, I think having some training and some teaching on that would be really useful*” (276–277). Participant F, who had previously attended a secular self-compassion training, was keen that something similar was offered to priests: *“…mindful self-compassion would be good in theology college and training? Yes! And that definitely wasn’t there*” (460–462). Adding, “*Yeah, go for it. Stick it in a module*” (471–4742). This is a novel finding that establishes evidence for the existence of interest in more self-compassion teaching in clergy training.

### Self-Compassion Clashes with Vocational Expectations of Self-Sacrifice

This second sub-category suggests another reason why self-compassion lacks theological coherency. It expressed a shared concern regarding a significant clash between the vocational call to self-sacrificing compassion for others, and the requirements of self-compassion to attend to one’s own well-being. Participant B articulated this tension clearly:

“*I think that’s where the guilt comes. Because we, you feel you do this because it’s a sacrifice, right? So…there is a sense of sacrifice to that because your life is there for others and there’s an important element to that and it almost can feel wrong sometimes when you’re putting yourself first.*” (497–501).

Illuminating the profound way self-sacrifice feels part of clergy vocation/calling (Edwards et al., 2022), this participant described what they felt was a constant ‘*Gethsemene experience’* (506), where there are always circumstances present or upcoming in ministry-life that feel deeply self-sacrificial (the garden of Gethsemene was where Jesus spent a final night in prayer before his crucifixion). Participant E extended this concern to parts of the world where Anglicans are being persecuted, and levels of self-sacrifice severe and traumatic. Where is a role for self-compassion in these situations, was their penetrating question. Noting the acute tension between giving potentially the greatest sacrifice possible or giving yourself compassion, this participant said: “*there is a sense in which depending on where you are, this can feel very first world*” (671).

### Self-Compassion Requires a Surrounding ‘Habitus of Compassion’

This category was constructed around the dataset’s most populous code ‘A habitus of compassion’: A supportive surrounding culture is key for clergy self-compassion. The term ‘habitus of compassion’ was used by Participant B to convey the felt need for clergy self-compassion to be nested within a wider culture of safety, congruent values and supportive care on an organisational level (i.e. The Church of England as a cultural body). Participant B was pinpointing the idea that important values/beliefs are created and sustained in organisations and other groups by an interplay between individual histories, beliefs and behaviours (personal habitus) of employees and that of the formal organisation (often referred to as the social ‘field’) (Bourdieu, 1995). This term habitus helps to highlight the insistent pattern in the data related to the inevitable relationality between individual priests practising self-compassion, and the wider contextual field of the Church and the impact of its culture and values on the ability of its clergy to live self-compassionately:

“*And so, like the classic ‘culture eats strategy for breakfast’, if there wasn’t a culture where we can be, where we can show compassion to each other, we show grace to one-another, then it’s harder to have self-compassion because you feel like you’re swimming upstream*” (Participant B, 603–606).

This participant was recognising the need for, as they termed it, a “*compassionate habitus*” (618) because otherwise self-compassion teaching/training could just become another onerous item on an endless to-do list within a wider culture that may be failing to provide structure and resources and care to help priests live more self-compassionately. As they explain:

“*I don’t think it’s just about training on it because it becomes like, oh, there’s another thing that we need to do as clergy, something else we need to look at when actually there needs to be a structure that enables that to happen*”? (Participant B, 309–311).

This demonstrates that self-compassion is understood by clergy to require others in the working environment to create space, opportunity and permission to take self-compassionate decisions, in short it cannot be entirely individualistic.

A perception that non-compassionate expectations/demands placed on clergy was a key factor mitigating against them being able to act self-compassionately was also seen across the dataset. It was suggested that excessive work-related demands lacking in empathic compassion escalate clergy feelings of failure, self-criticism and exhaustion, all of which clash with the ability to hold compassion for self and maybe even for others: *“…the actual practical outworking of that is quite hard with the demands that are placed on us*” (Participant C, 221–222). Importantly, they went on to state that the church’s training for clergy on self-care and well-being simply does not align with the pressures placed on them:

“*I found it quite a paradox really, because there is [training] in the preparation for theological college, in self-care and well-being. But then, alongside that you’re just hit with all these deadlines and expectations… You’re going, OK, how do I, how do I hold ‘this’ with ‘this’? Because what you’re telling me I’ve got to do, just doesn’t fit*” (377–382).

Furthermore, as Participant C noted (751–754) pressures don’t just come from institutional demands, for some it can also come from parishioners who impose impossibly high expectations on their priests. In this way, clergy can be vulnerable to an excess of pressure not only from colleagues, but from those they seek to serve, not unlike those in other caring professions such as health and social work sectors.

The data repeatedly suggested that, in places, the culture of the organisation, at local and wider levels, reduces the capacity for clergy to engage with self-compassion; however, conversely, where teams and colleagues display kindness and compassion, the capacity to cultivate self-compassion is tangibly enhanced: ‘*I’ve moved from a very dysfunctional team to an incredibly supportive team and that’s really helped with the whole compassion and self-compassion*” (Participant C, 775–776). In these ways, the findings point to some key differences in how clergy perceive and experience self-compassion versus compassion for others, as well as highlighting deep tensions for priests between the desire to cultivate self-compassion and their calling to self-sacrificial service.

## Discussion

The findings provided insights into clergy understanding and experience of compassion *(Compassion is highly valued and deeply rooted in theology)* and self-compassion *(Self-compassion is primarily understood as practical self -care).* However, they also worked synergistically: by comparing key insights from both categories, new information regarding the relative lack of theological depth ascribed to self-compassion could be contrasted with the theological validity and richness ascribed to compassion for others. This identification of a key difference in the way compassion for others and compassion for self is viewed by Church of England clergy is novel and raised the question of possible impacts of a relative lack of bible/faith-based motivation for self-compassion.

### Findings in Relation to Locus of Control

This finding could be partially explained by the long-standing psychological theory of Locus of Control (Rotter, 1966). The theory posits that people have varying degrees of belief in how much control they can exert on event outcomes in their lives (Rotter, 1966; Verma & Shah, 2017). The theory conceptualises Locus of Control as a continuum from internal to external locus of control along which those with strong internal Locus of Control believe they have power/agency to affect outcomes, whereas those with external Locus of Control believe that powerful forces outside of themselves (authoritative people/entities, luck or chance for example) are the arbiters of outcomes in their lives (Verma & Shah, 2017). Research suggests that Locus of Control has considerable impact (albeit concomitant with many other factors) on attitudes, behaviours and motivation (Verma & Shah, 2017). Internal Locus of Control has been associated with many markers of well-being and adaptive health behaviours (Gore et al., 2016; Klonowicz, 2001).

The data suggests a strong external Locus of Control for clergy, at least in relation to compassion, in which the outcome for certain behaviours/practices may, to some degree, be located externally in the promises and commands of God. This specific type of Locus of Control is sometimes referred to as ‘God-control’ (Jackson & Coursey, 1988), and there is no consensus on where this sits on the Locus of Control continuum, partly because personal and doctrinal understandings of faith are themselves so varied (Wong-McDonald & Gorsuch, 2004). While the present study does not seek to answer these questions, the adjunct Locus of Control literature may provide psychological insights regarding how clergy are likely to view the validity and efficacy of self-compassion practices in comparison with obeying the Divine command to show compassion to others and/or receiving compassion directly from God.

The research of Wong-McDonald and Gorsuch (2004) suggested that Locus of Control in God, particularly surrender to God, was associated with effective coping and spiritual well-being, whilst practices independent of the self-God relationship were not as helpful for well-being. This needs to be investigated further to understand the most effective ways to offer self-compassion training to clergy. This is an important future avenue of research, because there may be less logical reasoning (or, indeed, funding justification) in providing existing secular self-compassion resources to a cohort that may not value them as much as faith-based resources.

### Current Provision and Acceptability of Self-Compassion Training for Clergy

The sub-categories of ‘*Self-compassion is not explicitly taught to clergy, but they would largely welcome it’* and *‘Self-compassion is primarily understood as practical self-care’* produced some novel information pinpointing a co-existing lack of formal teaching about self-compassion in Church of England clergy training, alongside enthusiastic interest from clergy to learn more about it. It may be that this indicates a curiosity to expand the understanding of self-compassion from practical self-care only to something broader and more theologically robust. A valuable extension of the study would be to examine how much value clergy perceive in placing self-compassion in a specifically Christian theological framework rather than a secular or more Buddhist-leaning one. The issue of clashing worldviews/religious beliefs for some clergy regarding the Buddhist basis of commonly used self-compassion material was brought up forcefully by the one participant who had done a self-compassion course: “*I’m not sure…some colleges would go for it as it sounds all a bit dodgy and heretical!*” (Participant F, 611–612). It might also be useful in the future to add an interview question asking if there existed a perception of a Buddhist bias in self-compassion programmes and literature, and what obstacles/problems wisdom from different traditions poses for Church of England clergy. The researchers were unable to find any existing psychological literature examining this issue for this specific Church of England clergy population. However, the acceptability for Christians of Buddhist material in mindfulness programmes (which include self-compassion elements) is commonly discussed informally on podcasts, blogs and increasingly rigorously now in books by Christian authors with a background in psychology and priesthood; thorough examinations of the topic by Bretherton et al. (2016), Lambert (2024), and others are helping to roadmap a detailed theological conceptualisation and adaptation of practices for mindfulness/compassion within a specifically Christian spirituality.

Research considering the acceptability of material from other wisdom traditions might not only be a worthwhile topic for Church of England clergy but could also be relevant to research currently being carried out regarding increasing of access to mindfulness and compassion programmes for special interest groups (Loucks et al., 2022; Timbers & Hollenberger, 2022; Williams & Birtwell, 2018). This relates back to a live debate in the field around the central concern of Crane et al. (2017) ‘s paper regarding the need to expand mindfulness interventions appropriately and usefully across different contexts and populations whilst preserving the conceptual integrity of the key material. Issues raised in the data therefore touched on both accessibility and acceptability of self-compassion training for this population, and these aspects will need to be addressed in future research.

### Tensions Between Self-Sacrifice and Self-Compassion

As the second sub-category identified, in order to adapt self-compassion resources for this clergy context, some attention is required around the extremely complex tension that exists for clergy between a vocational expectation of self-sacrifice (Dillen, 2017; Page, 2016) and the benefits of self-compassion now increasingly evidenced for well-being (Ferrari et al., 2019), even in high-demand roles (Garcia-Carmona et al., 2019). This finding may provide an empirical direction towards some useful future cross-field collaboration between theologians, psychologists and self-compassion training experts to understand more deeply how certain vocations/callings go beyond self-interest and even, in extremis, self-preservation—and how/if this can be reconciled with practicing self-compassion.

Clinton et al. (2024) suggests that secure self-esteem may be a factor which aids regulation of the pull towards self-sacrifice in high demand callings and the need for sustainable well-being (specifically the avoidance of exhaustion), but more research is required to verify this. It would be interesting to see whether the research by Sahdra et al. (2023) which looked at the need to secure ‘*self-other harmony in the relations between self-compassion, other-compassion and well-being’* (p.1997), would be applicable to those in this high-demand vocational setting for which putting others before yourself is the expectation. Whilst many secular jobs are vocational and may entail forms of self-sacrifice, for clergy there is a Divine mandate for self-sacrifice (modelled by the crucified saviour) which may be particularly compelling and complex to hold alongside self-compassion. Again, this is an important question raised by the data, for which there are no immediate answers concomitant.

### Workplace Culture and Self-Compassion

The third category brought to the fore new insights into how clergy both experience a lack of compassion in the institution of the Church of England at times, and tangible support and care at other times—revealing a mixed picture of the experience of workplace social support for this cohort. The clearly articulated need persisting through the dataset for a compassionate environment for clergy self-compassion to feel sustainable and useful, points towards a nuanced understanding of the inter-dependence and intrinsic relationality of compassion and self-compassion (Gilbert, 2015). It also underlines the necessity for ethical application of self-compassion training on order to avoid it becoming a source of harm to its recipients rather than benefit. Participant data suggested, with some persistence, that if self-compassion cultivation becomes a coping strategy taught to increase resilience but surrounding institutional flaws or oppressive cultural norms in the workplace environment are not addressed, then those interventions may in fact prop up rather than challenge organisational injustices. This has been something that has been levelled as a vulnerability of some mindfulness-based interventions in the literature (Walsh & Arnold, 2020). Strategies to enhance a secure and clergy-supportive ‘habitus of compassion’ within the Church of England are beyond the scope of this study, but the request by clergy to consider the culture of the wider workplace environment before implementing self-compassion training, was a valuable contribution of this category.

### Limitations and Future Research

This successful pilot study produced novel findings of interest regarding key aspects of how clergy regard and experience compassion for others and self. This warrants extension of the current pilot study to discover how self-compassion training could be most effectively delivered in the clergy context, and in what ways this might require systemic organisational change and theological applications. The limitations of this current study will help inform subsequent research plans.

Firstly, a key limitation was the use of inductive, Qualitative Content Analysis because it imposed restrictions on how much and which parts of the data could be used to produce the study findings. The quantitative step of creating categories from only the most frequent codes necessarily meant that some potentially interesting, even important findings never made it into the report—for example data suggesting the Buddhist quality of much self-compassion conceptualisation and training material might be considered heretical and therefore likely rejected by some quarters of the Church of England. This example suggests an important issue potentially affecting the uptake of self-compassion training in this context, but one that was rejected for key category formation because it only occurred once in the data. This was a down-side of the study design, and it may be useful in the future to replicate this study using a different type of analysis protocol which would avoid this problem.

Future research focussed on expanding findings around the current research question should consider analytical approaches such as Reflexive Thematic Analysis (RTA) (Braun & Clarke, 2019). This alternative analytical design would give greater freedom to work both more interpretively and with latent meanings, but also not be limited to working with frequency as a key operation for theme generation, thereby allowing researcher/s to use whatever data they deem most salient. Interpretative Phenomenological Analysis (IPA) (Alase, 2017) would allow a deeper and more extensive exploration of how individual clergy find meaning and make sense of the specific phenomenon of self-compassion, both personally and in their working ministry context. The inductive, reflexive researcher stance in IPA would allow a double hermeneutic (Joseph, 2014; Smith, 2004) wherein both participants and researcher co-interpret the data and potentially are able to form broader theories from specific experiences. Although both approaches have the merits discussed, on balance the specific ability of RTA to generate broader themes on an organisational and systemic level is more aligned to the purpose of investigating individual experience as it exists not in isolation but embedded within the complex pressures and dynamics of a large organisational culture. Continued development of our knowledge and understanding in this area would support a longer-term goal of creating a context-suitable training tool to improve the self-compassion resources of clergy and thereby support their well-being.

Secondly, the interviews did not explore if there was any previous exposure to self-compassion literature/training programmes. This might be a useful future addition since it would bring clarity to how much, and what source of knowledge clergy have about self-compassion as a construct/concept prior to the study, and if/how this affects their perception of self-compassion. Further interview questions elucidating length of ministry service, age, conservative/evangelical versus liberal theological beliefs, and size of parish would produce further demographic data from which interesting and significant patterns and associations may emerge. It should be noted that running a pilot study was useful in bringing these areas of limitation to the fore, and guiding adaptations for high quality data collection and analysis in subsequent studies.

Future research may also wish to expand future sample groups in terms of both greater size and greater demographic diversity. This increased sample diversity would aid transferability, and in time extending the clergy sample beyond the Church of England denomination would reveal similarities and differences within different church traditions.

## Conclusion

At the very heart of the clergy vocation, as mentioned several times by participants, is to love God, neighbour and self (Matthew 22:37), a secure mandate and foundation for the relevance of self-compassion cultivation for this population. Examining more comprehensively the acceptability of, and access to self-compassion training/teaching in context-sensitive ways may help to address the desire clergy have to increase their self-compassion, simultaneously building a more compassionate culture within the Church of England.
